# Acute Effects of Non-Oil-Seed Pulses on Parameters of Cardiometabolic Health: A Systematic Review of Human Intervention Studies

**DOI:** 10.1093/nutrit/nuaf190

**Published:** 2025-11-29

**Authors:** Tim B Schiemann, Christina Diekmann, Sarah Egert

**Affiliations:** Institute of Nutritional and Food Sciences, Nutritional Physiology, University of Bonn, 53115 Bonn, Germany; Institute of Nutritional and Food Sciences, Nutritional Physiology, University of Bonn, 53115 Bonn, Germany; Institute of Nutritional and Food Sciences, Nutritional Physiology, University of Bonn, 53115 Bonn, Germany

**Keywords:** systematic review, postprandial metabolism, non-oil-seed pulses, legumes, glycemia, insulinemia, lipemia, hunger, satiety

## Abstract

**Background:**

Non-oil-seed pulses offer a plant-based source of dietary protein and further nutritionally valuable nutrients such as essential micronutrients and dietary fiber, making them a key component of sustainable diets emphasizing plant protein. They are also a relevant dietary source of carbohydrates with a low glycemic index.

**Objective:**

This systematic review aimed to evaluate the acute effects of a variety of non-oil-seed pulses on various parameters of cardiometabolic health, hunger, and satiety.

**Methods:**

In this systematic review, a literature search in the PubMed, Cochrane Library, and Scopus databases was conducted, and 40 human intervention studies were identified that investigated the effects of non-oil-seed pulses on postprandial metabolic events.

**Results:**

Most of the articles in this review reported that non-oil-seed pulses cause lower glucose (28 of 40 studies) and insulin (16 of 24 studies) responses than other starchy foods in the control groups (mainly wheat-flour–based products or white rice). These results were reported for a variety of non-oil-seed pulses and pulse products. Additionally, 3 of 5 studies found a significantly reduced feeling of hunger following meals enriched with pulses compared with the control meal, while the remaining 2 studies found a nonsignificant reduction. Although the studies examining satiety and fullness found increased satiation following pulse-enriched meals, only 1 of 6 studies found a significantly greater increase in satiety compared with the control, and 3 of 6 studies found a significantly greater increase in fullness. Other study variables, such as parameters of lipid metabolism and vascular function, have only been investigated in a few studies, and those studies mostly reported no significant effects.

**Conclusion:**

This systematic review confirmed that non-oil-seed pulses can attenuate postprandial glycemia and lipemia and can therefore be classified as low-glycemic food. Future human intervention studies should focus on the acute and chronic effects of non-oil-seed pulses on further health-related parameters, such as inflammatory markers and vascular function.

**Systematic Review Registration:**

PROSPERO registration No. CRD42023471539.

## INTRODUCTION

Legumes belong to the *Fabaceae* family and can be split into two subcategories: oil-seed legumes and non-oil-seed legumes. Non-oil-seed legumes are further divided into pulses and freshly harvested legumes. Pulses or ‘non-oil-seed pulses’, such as lentils, chickpeas, and dry beans, remain on plants to dry before being harvested and are a low-glycemic carbohydrate source. In addition, they are rich in protein, dietary fiber, vitamins (eg, folate), minerals, trace elements (eg, iron and magnesium), and selected phytochemicals (eg, flavonoids, phenolic acids).^1–3^ The protein content of legumes makes them the main plant-based protein source in the Planetary Health Diet.^4^ However, the nutrient content of different legumes varies. Table 1^5^ summarizes the nutrient composition (macronutrients and selected micronutrients) of various pulses.

**Table 1.** Energy and Nutrient Content of Selected Pulses (per 100 g Dried Product)

	Kidney beans	Split peas, green	Chickpeas	Lentils	Lupins
Energy (kcal)	337	364	378.0	352	371
Protein (g)	22.5	23.1	20.5	24.6	36.2
Cysteine (g)	0.3	0.3	0.3	0.3	0.5
Isoleucine (g)	1.0	1.0	0.9	1.1	1.6
Leucine (g)	1.8	1.7	1.5	1.8	2.7
Lysine (g)	1.8	1.8	1.4	1.7	1.9
Methionine (g)	0.3	0.2	0.3	0.2	0.3
Phenylalanine (g)	1.2	1.2	1.1	1.2	1.4
Threonine (g)	1.0	0.8	0.8	0.9	1.3
Tryptophan (g)	0.3	0.2	0.2	0.2	0.3
Valine (g)	1.2	1.0	0.9	1.2	1.5
Carbohydrates (g)	61.3	61.6	63.0	63.4	40.4
Fat (g)	1.1	3.9	6.0	1.1	9.7
SAFAs (g)	0.2	0.4	0.6	0.2	1.2
MUFAs (g)	0.1	0.6	1.4	0.2	3.9
PUFAs (g)	0.6	1.0	2.7	0.53	2.4
Dietary fiber (g)	15.2	22.2	12.2	10.7	18.9
Folate (µg)	394	15	557	479	355
Iron (mg)	6.7	4.7	4.3	6.5	4.4
Zinc (mg)	2.8	3.5	2.8	3.3	4.8
Magnesium (mg)	138	63	79	47	198
Calcium (mg)	83	46	57	35	176
Phosphorus (mg)	406	334	252	281	440

Source: United States Department of Agriculture (USDA), FoodData Central.^5^ MUFAs, monounsaturated fatty acids; PUFAs, polyunsaturated fatty acids; SAFAs, saturated fatty acids.

**Figure 1.**
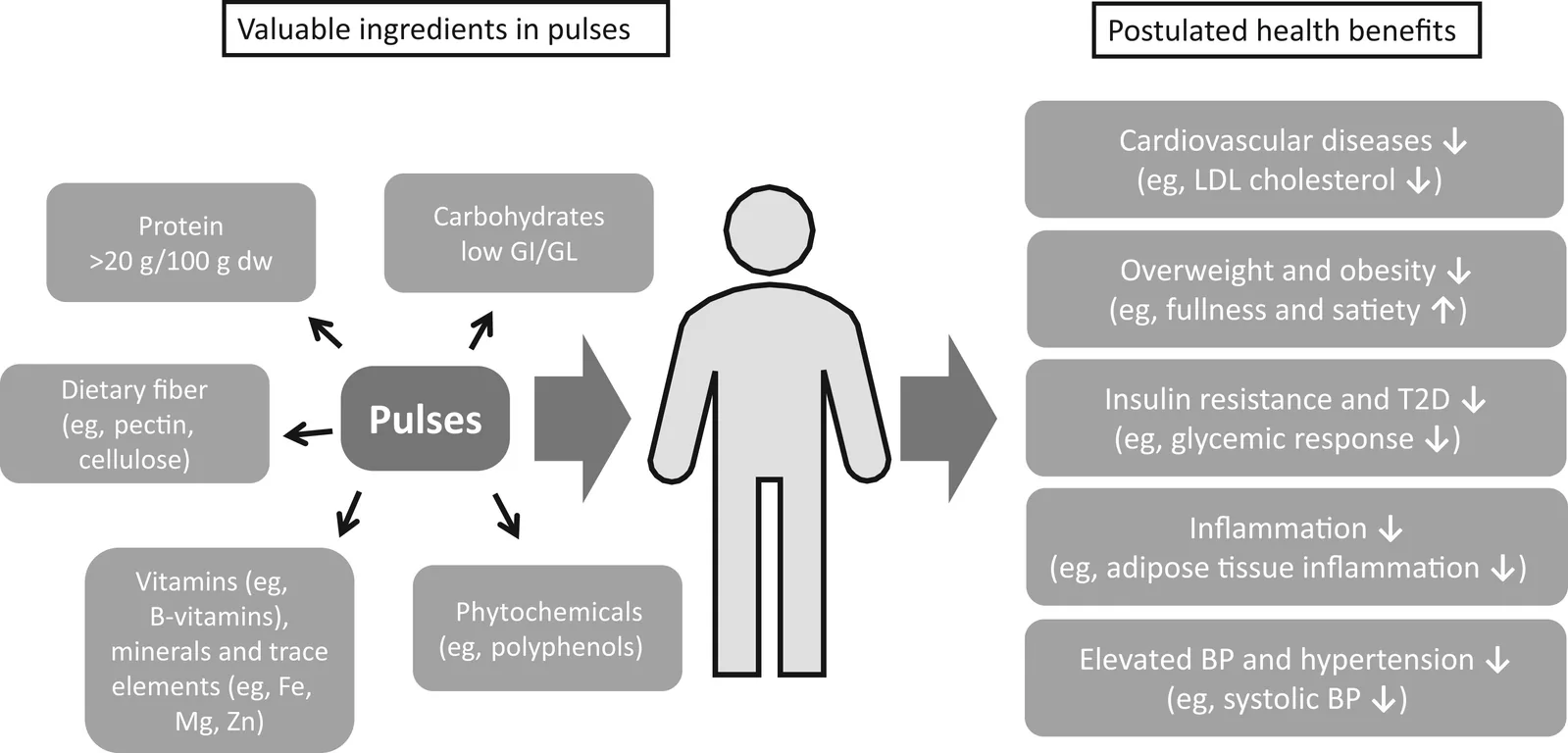
Composition and Postulated Health Benefits of Regular Pulse Consumption^1^^,^^8^^,^^18^^,^^19^ BP, blood pressure; CRP, c-reactive protein; dw, dry weight; Fe, iron; GI, glycemic index; GL, glycemic load; LDL, low-density-lipoprotein cholesterol; Mg, magnesium; T2D, type 2 diabetes; Zn, zinc.

Epidemiological data suggest that higher intake of legumes and especially pulses is associated with various health benefits, leading to reduced healthcare costs.^6–8^ The health benefits of legume consumption in general or in exchange for animal foods include reduced incidences of type 2 diabetes mellitus,^8^ cardiovascular diseases,^8–11^ and all-cause mortality.^9^^,^^12^ Owing to these health-promoting characteristics, many scientific dietary guidelines promote regular consumption of legumes, although the intake recommendations vary widely between countries.^13^^,^^14^ Furthermore, some meta-analyses of chronic intervention studies have concluded that regular consumption of pulses alone or in the context of a low-glycemic-index or fiber-rich diet improves markers of glycemic control^15^ and can lower total and low-density-lipoprotein (LDL) cholesterol concentrations.^16^^,^^17^ The postulated health benefits of regular pulse consumption are summarized in Figure 1.^1^^,^^8^^,^^18^^,^^19^

In addition to the chronic effects associated with regular pulse consumption, acute modulation of the postprandial response to pulse-enriched meals is of great interest. The postprandial state is characterized by increases of glucose (glycemia), insulin (insulinemia), and lipid (lipidemia) concentrations in the blood, resulting in a short-term imbalance between oxidative metabolic stress and antioxidant defense. Postprandial oxidative stress is associated with postprandial low-grade inflammation and endothelial activation.^20^ The accumulation of postprandial metabolic processes alongside a hyperenergetic diet appears to be associated with an increased long-term risk of the development of cardiometabolic and neurodegenerative diseases,^21^^,^^22^ with postprandial lipidemia,^23^ glycemia,^24^ and inflammation^25^ representing independent risk factors. Thus, strategies to weaken the postprandial response need to be developed.

A food group that might lead to a weakened postprandial response is non-oil-seed pulses. In particular, their low glycemic index and high dietary fiber and protein contents may reduce the glucose and insulin responses. This attenuated postprandial glucose response was demonstrated by 2 meta-analyses and systematic reviews focusing on lentils^26^ and chickpeas^27^ in acute dietary intervention trials. Additionally, the protein and dietary fiber content of pulses may lead to decreased postprandial hunger sensations and increased satiety compared with other starchy foods like grain products.^28^ Therefore, incorporation of non-oil-seed pulses into a diet may be beneficial in the context of the worldwide increases in overweight and obesity, because increased satiation may reduce food intake and therefore prevent body weight gain.^29^ A meta-analysis of randomized controlled trials with a duration of more than 3 weeks reported a modest weight-loss effect upon inclusion of pulses in a diet.^30^ The acute effects of pulses on hunger and satiety-related outcomes were summarized by a meta-analysis in 2014, which reported a greater incremental area under the response curve (iAUC) of satiety without affecting second meal food intake compared with the control.^31^ In addition to literature reviews focusing on satiety ^31^ and glycemic responses to lentils^26^ and chickpeas,^27^ reviews summarizing the effects of all non-oil-seed pulses on a variety of postprandial outcome parameters are needed.

Therefore, this systematic review aimed to summarize the current findings of acute human intervention studies investigating the effects of non-oil-seed pulses on metabolic parameters (eg, glucose, insulin, glucagon-like peptide-1 [GLP-1], and triglycerides [TAGs]), vascular function, endothelial activation (eg, soluble endothelial adhesion molecules), and parameters of hunger and satiety.

## METHODS

### Protocol and Registration

This systematic review was prospectively registered at PROSPERO (CRD42023471539). We followed the PRISMA statement 2020.^32^

### Literature Search and Study Selection Process

A systematic literature search was conducted in the PubMed, Cochrane Library, and Scopus databases between August and November 2023. An update search was carried out in November 2024. The search term (beans OR bean OR lentils OR lentil OR chickpeas OR chickpea OR peas OR pea OR legume OR legumes OR leguminous OR lupin OR lupins OR dietary pulses OR dietary pulse) AND (glucose OR insulin OR diabetes OR triglycerides OR cholesterol OR oxidative status OR inflammatory OR inflammation OR endothelial OR vascular OR body weight OR overweight OR obesity OR blood pressure OR hypertension OR metabolic syndrome OR cardiovascular) AND human AND (trial OR study) AND (postprandial OR acute) was used to identify relevant studies. Two authors independently screened the literature. Discrepancies were resolved through discussions within the review team. The research question was formulated using the PICOS criteria (participants, interventions, comparisons, outcomes, and study design) to ensure a systematic and comprehensive approach. A detailed description of the PICOS criteria used in this study as well as the derived inclusion and exclusion criteria is provided in Table 2. Studies were included if they were of randomized crossover or parallel design and analyzed postprandial responses in humans; if participants consumed at least one meal enriched with non-oil-seed pulses and one control meal, either without non-oil-seed pulses or another type of legume; if postprandial glucose, insulin, TAGs, blood biomarkers of inflammation, or endothelial function was analyzed periodically at regular time-intervals; and if the paper was written in English. Studies were excluded if non-oil-seed pulses were served as shakes; if only fractions of pulses were used; or if the legume part of the meal was administered as a capsule. Furthermore, studies investigating hospitalized patients were excluded. Additionally, studies where only the conference abstract was available were excluded.

**Table 2.** PICOS Criteria for Inclusion and Exclusion of Studies

Parameter	Inclusion criterion	Exclusion criterion
Participants	Adults	Children or hospitalized patients, animal studies
Interventions	Non-oil-seed pulses	Non-oil-seed pulses served as shakes, only fractions of pulses used, or the legume part of the meal administered as a capsule
Comparisons	Non-pulse food items or non-oil seed pulses other than those used in the intervention group	Food items served as shakes or administered as a capsule
Outcomes	Metabolic parameters (eg, glucose, insulin, triglycerides) measured periodically at regular intervals	Studies without extractable numerical data for metabolic parameters
Study design	Randomized controlled trials with a postprandial design	Cohort studies, cross-sectional studies, longitudinal studies, chronic intervention studies

### Data Extraction and Quality Assessment

The data were extracted by one investigator and checked for accuracy by an independent second reviewer. The data extracted from the included publications were author, year of publication, number of subjects, age and health condition of the population, study design, duration of the postprandial period, a brief description of the intervention arms and test meals, relevant outcomes (glucose, insulin, C-peptide, GLP-1, TAGs, total cholesterol [TC], LDL cholesterol, high-density-lipoprotein [HDL] cholesterol, amino acids, soluble intercellular adhesion molecule-1 [sICAM1], soluble vascular cell adhesion molecule-1 [sVCAM1], augmentation index [AIX], pulse wave velocity [PWV], ghrelin, peptide YY [PYY], cholecystokinin [CKK], hunger, satiety, fullness, appetite, and *ad libitum* food intake), and the main results for the respective outcomes.

The quality of the selected studies was assessed according to the Revised Cochrane Risk of Bias tool for randomized trials (RoB 2) with additional considerations for crossover trials, considering the randomization process, period, and carryover effects, deviations from the intended intervention, missing outcome data, measurement of the outcome, and selective reporting.^33^ Each domain was rated as having low risk, some concerns, or high risk of bias. The overall quality of each study was determined to be good if the study scored low risk for all domains, fair if the study scored some concerns for one or more domain, and poor if the study scored high risk for one or more domain. The risk of bias was assessed by one investigator and checked for plausibility by an independent second reviewer. Discrepancies were resolved by a discussion with a third reviewer.

## RESULTS

**Figure 2.**
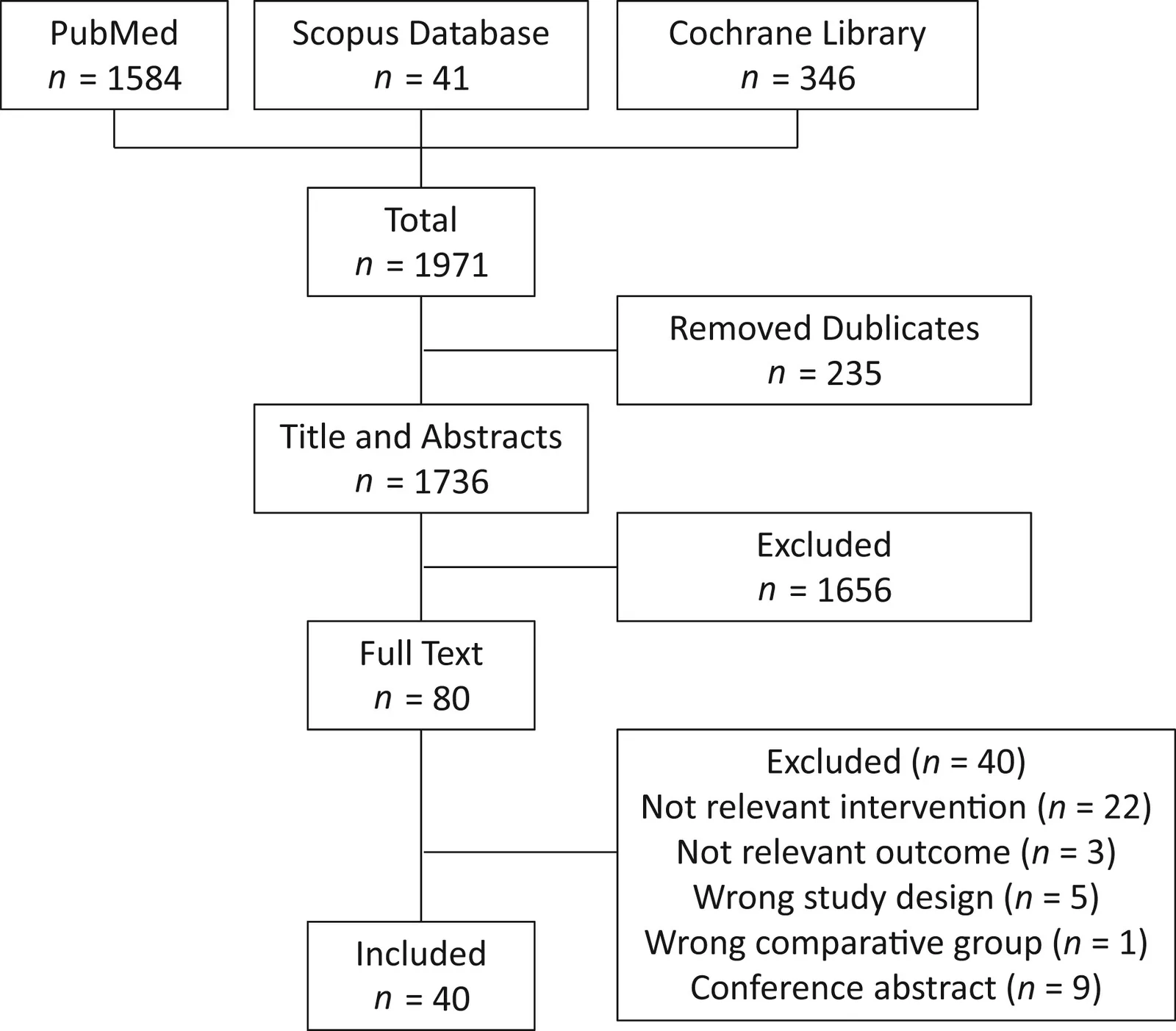
Flowchart of the Article Search and Selection Process

The systematic literature search in the PubMed database identified 1584 publications. Additionally, 346 studies were found in the Cochrane Library database and 41 studies were found in the Scopus database. After removal of duplicates, 1736 studies were screened by their title and abstract. Of these, 1656 studies were excluded because they did not fulfill the inclusion criteria and/or fulfilled at least one exclusion criterion. After examining the full texts of the remaining 80 studies, 40 publications were included in this review (Figure 2).

The included studies were published between 2001 and 2024. Thirty-five studies included apparently healthy subjects, whereas 3 studies included patients with type 2 diabetes mellitus.^34–36^ One study recruited overweight subjects,^37^ while another study included participants with metabolic syndrome.^38^ The mean age of participants ranged from 20^39^ to 66.4^34 ^years. All studies were crossover trials. A postprandial time period of 120–420 minutes was observed. Some studies investigated the effects of bread, pasta, or muffins enriched with chickpeas,^40–44^ yellow peas,^43^^,^^45–48^ lupins,^49–52^ beans,^43^^,^^53–57^ or lentil flour^43^^,^^56^ or a combination of different legume flours,^58^ whereas most of the other studies examined the effects of whole non-oil-seed pulses.^34–36^^,^^38^^,^^54^^,^^59–69^ Singh et al compared a snack based on bengal gram (chickpea) with a snack based on black gram (urad bean).^39^ Another intervention study investigated the effects of bean flakes.^70^ Two further studies used a hummus snack based on chickpeas as the intervention meal.^37^^,^^71^ Anderson et al used differently prepared pulses, including whole, pureed, and powdered forms of navy beans, lentils, and chickpeas, in 3 individual trials.^66^ Alshaalan et al compared a hummus made from intact chickpea cells with a hummus made from ruptured chickpea cells.^72^ In the pilot study by Reverri et al, a fiber-matched meal and an antioxidant capacity–matched meal were used as comparators.^38^ Another study conducted 3 trials, 2 of which used green or red lentils in a chili or soup meal and one of which used muffins enriched with lentil flour.^73^ The amount of legumes per test meal varied from about 10 g of lupin flour in a lupin bread^49^ to 406 g of canned yellow peas.^67^ The energy content of the meals ranged from approximately 190 kcal^43^ to 933 kcal.^38^

The comparator to the non-oil-seed pulse intervention was white bread in 11 studies,^34^^,^^37^^,^^40^^,^^42^^,^^47^^,^^49–51^^,^^55^^,^^58^^,^^61^ white rice in 6 studies,^36^^,^^45^^,^^54^^,^^63^^,^^69^^,^^70^ wheat pasta in 7 studies,^46^^,^^52^^,^^53^^,^^59^^,^^60^^,^^64^^,^^67^ potatoes in 3 studies,^35^^,^^41^^,^^64^ and a muffin in 1 study.^56^ Another study used the traditional Greek meals trahana and halva as control meals.^62^ Further comparators were whole wheat bread,^44^^,^^66^ a granola bar,^71^  *Lemna minor* (duck wheat),^65^ and corn flour.^43^ Rossukon et al compared a cracker enriched with white kidney bean flour with a wheat cracker, a cracker enriched with cashew nut flour, and a cracker enriched with almond flour.^57^ The study by Schopen at al had two arms in addition to the lupin-enriched intervention. The first comparator was supplemented with whey, while the reference meal included a pasta sauce with neither whey nor lupin flour supplementation.^52^ Marinangeli et al used whole wheat flour in the comparator groups.^48^ Chamoun et al, who conducted 3 trials within their study, used different comparators in each trial. The first trial used wheat flour, the second trial used white rice, and the third trial used potatoes.^73^ Additionally, 18 studies had more than one intervention arm with a non-oil-seed-pulse–enriched meal.^34–36^^,^^39–43^^,^^45^^,^^54–56^^,^^59–61^^,^^66^^,^^68^^,^^73^ Four studies did not conduct the investigation in a fasted state but provided a standardized breakfast a few hours before the actual intervention^53^^,^^59^^,^^66^ or a standardized breakfast and a lunch before a snack intervention.^71^ A summary of the included studies and their results can be found in Supplementary Table 1.

### Impact of Non-Oil-Seed Pulses on Postprandial Glycemia

All 40 studies analyzed the postprandial blood glucose concentration. Most (*n* = 28) of the studies reported significantly lower iAUCs of glucose following at least one of the legume meals than following the control meals.^34–37^^,^^40^^,^^41^^,^^43^^,^^45^^,^^46^^,^^48^^,^^50–52^^,^^54–56^^,^^58^^,^^59^^,^^61–68^^,^^71^^,^^73^ The remaining studies did not find significant effects on the iAUC of glucose.^38^^,^^39^^,^^42^^,^^44^^,^^47^^,^^49^^,^^53^^,^^57^^,^^60^^,^^69^^,^^70^^,^^72^ Schopen et al reported a significant reduction in the iAUC of glucose for the lupin meal compared with the reference pasta meal, but found no significant effect compared with the whey meal.^52^ The results are shown in Supplementary Table 1.

While some studies investigating the glycemic effect of different non-oil-seed pulses reported a significant reduction^34^^,^^35^^,^^39–41^^,^^45^^,^^56^^,^^68^ or no significant reduction^42^^,^^59^^,^^72^ in postprandial glucose for all legumes investigated, others reported different results regarding the effects of different pulses on postprandial glucose concentrations. For example, Anderson et al reported a significant reduction in the mean postprandial glucose concentration following different lentil and chickpea meals, but did not observe a significant effect following consumption of navy beans.^66^ Johnston et al reported a significantly smaller increase in glucose compared with the control following consumption of the pinto bean and chickpea snacks only, but reported no significant effects following consumption of the split yellow pea and green lentil snacks. Consumption of the whole yellow pea snack even led to an increased glucose response compared with the control. These differences resulted in a significantly lower glucose response to the pinto bean and chickpea snacks than to the yellow pea snack.^43^ Partly consistent with these results are the data reported by Mollard et al who observed a significantly lower iAUC of glucose following consumption of chickpeas, navy beans, and lentils, but not following consumption of yellow peas.^59^ Another study that reported differing results between different non-oil-seed pulses was by Thompson et al, who reported a significantly lower iAUC of glucose after the consumption of pinto beans and black beans, but not of kidney beans, compared with the control.^36^ Winham et al reported a significantly lower glucose concentration after consumption of chickpeas compared with the control meal, but observed no significant effect after consumption of black beans.^54^ The same author group conducted a further study investigating the effects of consumption of black bean pasta and whole black beans on postprandial glucose concentrations. Only consumption of whole black beans, not black bean pasta, resulted in a significantly lower glucose response.^55^ Similar to these results, a study using whole yellow pea flour reported a significant reduction of the postprandial iAUC of glucose for the banana bread and biscotti intervention arms compared with the control, while the iAUC of glucose was significantly increased after consumption of yellow pea pasta compared with whole wheat pasta.^48^ In a trial using muffins enriched with lentil flour, Chamoun et al reported significantly reduced postprandial glucose concentrations following consumption of the muffin enriched with red lentil flour, but not of the muffin enriched with green lentil flour, compared with the control.^73^

### Impact of Non-Oil-Seed Pulses on Postprandial Insulinemia

Similar to the effects on glucose levels, most studies (16 of 24) reported a significant lowering effect of at least one of the investigated pulse meals on postprandial insulin concentrations.^34^^,^^35^^,^^37^^,^^38^^,^^40^^,^^43^^,^^45^^,^^46^^,^^50–52^^,^^55^^,^^58^^,^^61^^,^^72^^,^^73^ Six studies reported no significant change in the insulin response compared with the control.^47^^,^^49^^,^^53^^,^^54^^,^^69^^,^^70^ Shopen et al reported a significantly lower insulinemic response following the lupin-enriched meal compared with the whey meal, but observed no significant change compared with the meal lacking lupin and whey supplementation.^52^ Johnson et al reported a significantly higher insulin response after consumption of a bread enriched with chickpea flour than after consumption of a standard white bread.^42^ Compared with duck wheat (*Lemna minor*), consumption of peas resulted in significantly higher insulin concentrations at various time points in the postprandial state.^65^

While 3 studies investigating different non-oil-seed pulses reported the same effects for all intervention arms compared with the control,^34^^,^^35^^,^^54^ some studies (*n* = 7) reported various results regarding the effects of different pulses on postprandial insulin concentrations.^40^^,^^42^^,^^43^^,^^45^^,^^55^^,^^72^^,^^73^ Bajka et al reported significantly lower insulin concentrations than the control after consumption of the bread enriched with 60% chickpea flour, but found no significant difference in the insulin response after consumption of the bread enriched with 30% chickpea flour compared with the control.^40^ Another study using chickpea and extruded chickpea breads observed significantly higher insulin concentrations after consumption of the chickpea bread compared with the control, while there were no significant differences after consumption of the extruded chickpea bread compared with the control.^42^ Johnston et al reported different results regarding the insulin response following the consumption of different legume-enriched snacks. While consumption of the pinto bean and chickpea snacks resulted in significantly lower iAUCs of insulin than the control, consumption of the whole yellow pea and green lentil snacks had no significant insulin-lowering effect compared with the control. Consumption of the split yellow pea snack even resulted in higher insulin concentrations than the control. Compared with the whole yellow pea and split yellow pea snacks, consumption of the pinto bean snack resulted in a significantly lower iAUC of insulin.^43^ Winham et al reported a significantly lower postprandial insulin response following the consumption of whole black beans compared with the control, but observed no significant effects following consumption of black bean pasta.^55^ A further study comparing dehulled and unshelled yellow pea pasta with white rice reported a significantly lower iAUC of insulin following consumption of the unshelled yellow pea pasta, while consumption of the dehulled yellow pea pasta resulted in a higher insulin response than the control.^45^ Consumption of a hummus made from intact chickpea cells resulted in a significantly lower iAUC of insulin than consumption of a hummus made from ruptured chickpea cells.^72^ In the trial investigating muffins enriched with lentil flour, Chamoun et al reported significantly reduced postprandial insulin concentrations following consumption of the muffin enriched with green lentil flour, but not of the muffin enriched with red lentil flour, compared with the control.^73^

### Impact of Non-Oil-Seed Pulses on C-Peptide

Three studies investigated the effects of non-oil-seed pulses on C-peptide,^40^^,^^41^^,^^53^ 2 of which reported reduced postprandial concentrations compared with control meals.^40^^,^^41^^,^^53^ Bajka et al reported a significantly lower iAUC of C-peptide after consumption of the bread enriched with 60% chickpea flour compared with the white bread control.^40^ Another study investigating the effects of different chickpea meals compared with a mashed potato meal on the postprandial C-peptide response reported significantly lower iAUCs for all chickpea treatments.^41^

### Impact of Non-Oil-Seed Pulses on GLP-1

Three studies investigated the effects of non-oil-seed pulses on the incretin GLP-1^40,.41,53^ Bajka et al reported a significantly higher AUC after consumption of the bread enriched with 60% chickpea flour compared with the white bread control.^40^ In contrast, another study investigating the effects of different chickpea meals (compared with a mashed potato meal) on the postprandial GLP-1 response reported significantly lower AUCs for all chickpea treatments.^41^ Chan et al did not observe any significant effects of the legume intervention on the GLP-1 concentrations.^53^

### Impact of Non-Oil-Seed Pulses on Postprandial Lipemia

Five studies investigated the effects of non-oil-seed pulses on postprandial lipemia.^34^^,^^38^^,^^61^^,^^69^^,^^70^ Nestel et al and Bourdon et al did not find significant acute effects of chickpeas or bean flakes on TAGs, LDL cholesterol, or HDL cholesterol.^61^^,^^70^ While Nestel et al investigated the effect of high-fat meals containing more than 50 g of fat on postprandial metabolism,^61^ Bourdon et al used lower amounts of fat (approximately 19 g) in their test meals.^70^ Clark et al reported no significant effects of different beans on TAGs, TC, or HDL cholesterol. However, the study team found significantly lower LDL cholesterol concentrations 6 hours postprandially after consumption of black beans than after consumption of white rice.^69^ No information on the fat content of the meals was provided. A further study using meals containing 25 g fat reported no significant differences in postprandial TAGs after a black bean meal compared with a fiber- or antioxidant capacity–matched meal.^38^ Olmedilla-Alonso et al reported a significantly lower iAUC of TAGs after the almonga bean meal compared with white rice, but found no difference in the change of the TAG concentration after the curruquilla bean meal compared with the control.^34^ In this study, approximately 20 g fat was ingested.

### Impact of Non-Oil-Seed Pulses on Postprandial Vascular Function and Endothelial Activation

Three studies investigated the effects of non-oil-seed pulses on parameters of vascular function and/or endothelial activation.^38^^,^^64^^,^^69^ While Zurbau et al reported a significant reduction of the AIX after the chickpea meal at 4 hours postprandially compared with the control,^64^ another study investigating the influence of different beans on the AIX did not find any significant effects.^69^ Clark et al reported significantly lower PWVs at 2 hours after consumption of kidney beans and pinto beans than after consumption of navy beans. The PWV was significantly lower after the black bean meal than after the pinto bean meal at 6 hours postprandially.^69^ In the study by Reverri et al the postprandial concentrations of sICAM1 und sVCAM1 were significantly higher after the black bean meal compared with the control.^38^

### Impact of Non-Oil-Seed Pulses on Postprandial Amino Acids

The two studies investigating the effects of legumes on postprandial amino acids reported significantly higher concentrations of total amino acids^40^^,^^65^ and essential amino acids^65^ in the serum after the consumption of non-oil-seed pulses than after the control meal, but one study did not standardize the total amount of protein in the test meals.^40^

### Impact of Non-Oil-Seed Pulses on Hunger and Satiety

Twenty studies examined the effects of meals enriched with non-oil-seed pulses on subjective hunger and satiety as well as *ad libitum* food intake and hunger- and satiety-associated hormones or peptides.^40–43^^,^^46^^,^^47^^,^^49–51^^,^^53^^,^^55–57^^,^^59^^,^^60^^,^^62^^,^^63^^,^^67^^,^^70^^,^^71^

### Subjective Hunger and Satiety

Subjective hunger and satiety ratings were measured via visual analog scales. The hunger sensation during the postprandial period was assessed in 5 studies.^41^^,^^46^^,^^47^^,^^55^^,^^57^ Three of these studies reported significantly lower hunger following the non-oil-seed-pulse-enriched meals than after the control meal,^41^^,^^47^^,^^55^ while 2 studies reported no significant differences between the intervention and control groups.^46^^,^^57^ The results regarding postprandial appetite demonstrated a lower effect of legumes. While 2 studies reported significantly lower appetite ratings following the non-oil-seed-pulse–enriched meals,^55^^,^^56^ 7 studies did not report significantly different effects on appetite between the intervention and control groups.^43^^,^^53^^,^^59^^,^^60^^,^^62^^,^^67^^,^^71^ Eleven studies evaluated postprandial satiety or fullness. Four of these studies reported significantly higher satiety or fullness ratings compared with the control meal,^41^^,^^47^^,^^50^^,^^55^ whereas 7 studies did not find significantly different changes between the meals.^40^^,^^42^^,^^46^^,^^49^^,^^57^^,^^63^^,^^71^

### *Ad Libitum* Food Intake

Nine studies assessed *ad libitum* food and energy intake following legume-enriched or control meals in a second meal.^40^^,^^42^^,^^43^^,^^49^^,^^50^^,^^56^^,^^59^^,^^60^^,^^67^ Most of these studies (*n* = 7) did not report significant effects regarding *ad libitum* food intake in the second meal.^40^^,^^42^^,^^43^^,^^49^^,^^50^^,^^59^^,^^67^ Mollard et al reported a significantly lower *ad libitum* food intake in a second meal after the intervention meals enriched with lentils and yellow peas compared with the control. The effect was not significant for chickpeas.^60^ Another study found significant effects of different pulse-based functional muffins on *ad libitum* food intake compared with a control muffin. The pulse-enriched muffins resulted in significantly lower food intake 140 minutes after muffin consumption compared with the control meal.^56^ In one study, the participants were not given a standardized portion, but a meal with a standardized energy content per 100 g, which they were instructed to eat until they were comfortably full. While the chickpea and yellow pea meals tended to result in higher energy intake than the control meal, the lentil and navy bean meals resulted in significantly lower energy intake than the control meal.^59^ In the second meal, food intake did not significantly differ between the pulse and control meals.

### Cholecystokinin

The only study measuring the peptide hormone cholecystokinin (CCK) was conducted by Bourdon et al and reported significantly higher AUCs of CCK following the bean flake meal than following the rice meal.^70^

### Peptide YY

Two studies investigated the effect of non-oil-seed pulses on the peptide hormone PYY.^40^^,^^53^ Both studies had at least one intervention arm that resulted in a significantly higher postprandial PYY concentration compared with the control. In the study by Bajka et al, only consumption of the bread enriched with 60% chickpea flour resulted in a significantly increased PYY concentration compared with the control.^40^

### Ghrelin

The only study analyzing the hunger hormone ghrelin observed a significantly lower ghrelin concentration after consumption of a bread enriched with lupin flour than after consumption of the control bread.^51^

### Risk-of-Bias Assessment

**Figure 3.**
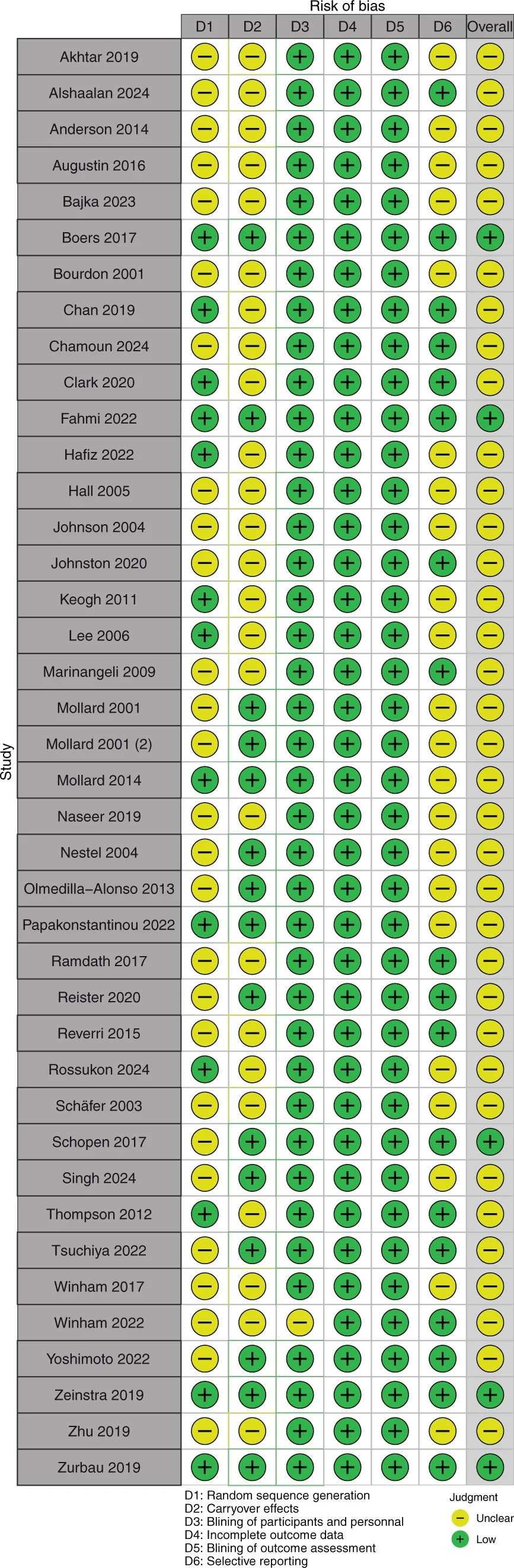
Risk-of-Bias Assessment. The figure was designed with robvis (visualization tool)^74^

**Figure 4.**
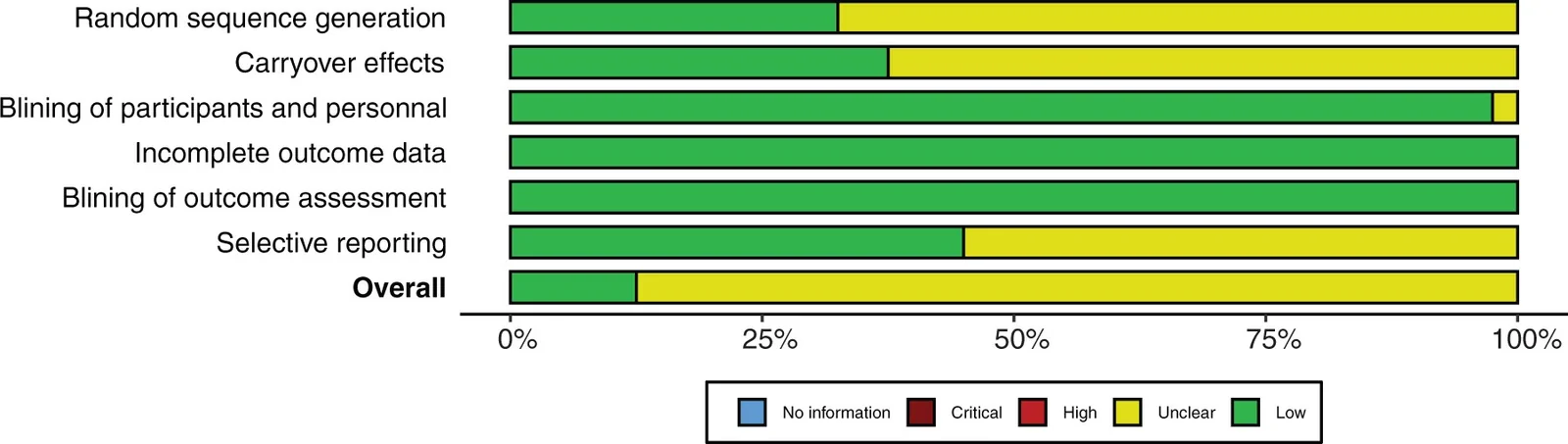
Summary Plot of Risk-of-Bias Assessment. The figure was designed with robvis (visualization tool)^74^

While 5 studies were graded as good quality because all domains were scored as having low risk of bias, the remaining 35 studies were graded as fair quality because at least one domain had some concerns regarding bias. The risk-of-bias assessment for the individual studies is shown in Figure 3. Figure 4 summarizes the risk of bias assessment of the included studies.

## DISCUSSION

In this systematic review, we aimed to summarize and analyze the existing evidence on the acute impact of non-oil-seed pulses on parameters of cardiometabolic health as well as on hunger and satiety in healthy adults and individuals with increased cardiovascular disease risk. This is the novelty of our review compared with the published reviews and meta-analyses, which are limited to investigations of the effects of individual legumes or specific outcome parameters.^15^^,^^26^^,^^27^^,^^31^ We specifically focused on studies that used a controlled postprandial design and administered the different pulses in “complete meals”.

Regarding glucose metabolism, most studies reported significantly lower postprandial glycemic (28 out of 40 studies) and insulinemic (16 out of 24 studies) responses after consumption of meals rich in non-oil-seed pulses, while some studies reported different results regarding the insulinemic response. The results regarding C-peptide are consistent with those regarding insulin. Other reviews focusing on the effects of single pulses (such as lentils^26^ or chickpeas^27^) on postprandial glucose metabolism concluded that legumes can lower the blood glucose level in patients with or without type 2 diabetes mellitus, which is in line with our results. While the protein content of most pulse-enriched meals exceeded that of the control meals, the observed reduction of the glucose response cannot be explained by the insulin-stimulatory effects of protein.^75^ This is evidenced by the decrease of the insulin concentration following most intervention meals compared with the control. A possible explanation is that a protein–starch interaction blocks digestive enzymes from accessing starch, which reduces degradation and absorption of glucose.^76^ Another explanation for the lower glycemic effects of the pulse meals might be the content of anti-nutritive compounds, because some of the polyphenols present in pulses inhibit α-glucosidase and α-amylase.^77–80^ This leads to reduced and slower digestion of dietary carbohydrates and therefore reduces postprandial glucose concentrations. In particular, flavonols like kaempferol and quercetin seem to contribute to the inhibitory activity on α-glucosidase.^77^ The inhibition is caused by binding of polyphenols to α-glucosidase.^81^ In addition to inhibition of enzymes responsible for carbohydrate degradation, polyphenols can modulate various pathways in glucose metabolism, as described in a recent review.^82^ While interactions with transporters like sodium/glucose cotransporter 1 (SGLT1) and glucose transporter type 2 (GLUT2) in the intestine result in reduced glucose absorption, glucose uptake in muscle cells is enhanced by activation of adenosine monophosphate activated protein kinase (AMPK), which promotes glucose transporter type 4 (GLUT4) translocation. Additionally, AMPK inhibits the gluconeogenic enzymes phosphoenolpyruvate carboxykinase and glucose 6-phosphatase, resulting in reduced hepatic glucose production.^82^ In summary, polyphenols in pulses may reduce plasma glucose concentrations through several pathways. The effects of pulses on glucose metabolism are mediated through multiple mechanisms, including delayed gastric emptying, reduced enzymatic hydrolysis of starch due to protein–starch and polyphenol–enzyme interactions, as well as the activation of key regulatory pathways such as AMPK and incretin signaling, which collectively modulate glucose uptake and insulin secretion.^76^^,^^77^^,^^81–83^ Various polyphenols influence these pathways in distinct ways; therefore, the diverse polyphenol composition of various pulses may also be associated with their varying effects on glucose metabolism.^77^^,^^79^^,^^82^ A further reason why the consumption of the meals with pulses led to reduced postprandial glucose concentrations might be the differing carbohydrate contents of these meals, because pulses contain lower amounts of available carbohydrates than other starchy foods such as wheat flour and white rice. While about half of the studies standardized for (available) carbohydrates, the other studies did not.^31^^,^^34^^,^^37^^,^^38^^,^^43^^,^^44^^,^^46^^,^^50^^,^^53^^,^^56^^,^^59^^,^^60^^,^^62^^,^^64^^,^^65^^,^^67^^,^^69–71^

Furthermore, the authors of a review investigating the effects of lentils on glucose metabolism stated that these effects were seen with different processing methods, because the reduction of the postprandial glucose concentration was maintained following the consumption of lentils in different forms compared with the control.^26^ This is consistent with the results of our review, because most of the included studies conducted with different intervention arms examining a variety of legume preparations reported significantly lower glucose and insulin responses following the pulse-enriched meals compared with the control. One exception might be the consumption of pasta made from pulses, because 2 studies^48^^,^^55^ reported significant lowering effects on postprandial glucose following the consumption of whole legumes and biscuits partly made from legume flour, but observed no effects for the pasta intervention. One of these studies^55^ reported the same differing effects between whole legumes and pasta made from legume flour on the insulin response, because consumption of the pasta had no significantly different effect on insulin concentrations compared with the control. Winham et al^55^ attributed the effect to intact cell walls in whole legumes and the increased surface area generated by food processing, facilitating greater access for human digestive enzymes. However, this explanation cannot account for the findings of other studies, which demonstrated significant lowering effects of processed legume flour interventions on postprandial glucose and insulin compared with the control. A possible reason for the difference in the glucose response between whole pulses and pasta made from pulses described by Mariangeli et al is starch gelatinization, which might improve starch hydrolyzation by digestive enzymes. This might lead to faster carbohydrate degradation and glucose absorption, resulting in a lower reduction of the glucose response compared with the control. Furthermore, an explanation might be the large surface area of pasta in boiling water. This might lead to greater destruction of anti-nutritive factors, which inhibit glucose absorption,^48^ compared with whole legumes or flour used in bread and pastries.

In addition to their effects on the postprandial glucose response, various phytochemicals found in pulses (eg, phytic acid, saponins, flavonols) have further postulated health benefits.^84^ Among those properties, antioxidant, antibacterial, and anticancer activities have been described.^85^ The anticancer activities are attributed to the anti-inflammatory activities, which are due to the regulation of inflammatory cytokines or mediators (eg, cyclooxygenase-2, tumor necrosis factor alpha, nuclear factor kappa).^85^^,^^86^ Furthermore, polyphenols, which can be found in pulses, are postulated to contribute to cardiovascular disease protection (eg, by decreasing systolic BP and by inhibiting cholesterol absorption, which may lead to reduced LDL cholesterol levels) (Figure 1).^19^^,^^85^^,^^87–89^

However, our results regarding postprandial lipemia are more heterogenous than those regarding postprandial glucose metabolism and indicate that non-oil-seed pulses do not significantly affect postprandial lipemia regardless of the amount of fat in the meal challenge and the legumes provided. Only one study analyzing postprandial TAG concentrations reported significantly different results following consumption of almonga and curruquilla beans. The AUC of TAGs was significantly lower only after the almonga bean meal compared with the bread.^34^ It could be assumed that differences in the phenolic content of compounds that act as lipase inhibitors between the bean variations might have led to differences in the TAG response.^79^

The 2 studies examining PWV or AIX as biomarkers of vascular function reported heterogenous results; only one study^64^ reported significant AIX-lowering effects at 4 h postprandially after the chickpea meal compared with the control. The study measuring PWV reported significant differences of PWV at 2 and 6 hours postprandially only between the different pulse interventions, but not compared with the control.^69^ The study analyzing sICAM1 and sVCAM1 reported significantly higher concentrations following the bean meal than following the isoenergetic fiber- and antioxidant capacity–matched control meals, which indicated higher postprandial endothelial activation and low-grade inflammation following the non-oil-seed-pulse–containing meals.^38^ While epidemiological data suggest that higher legume intake is associated with decreased concentrations of adhesion molecules,^90^ acute and chronic intervention studies examining the effect of legume intake on adhesion molecules are lacking. Reviews and meta-analyses summarizing the chronic effects of legume intake on inflammatory markers found no significant effects on C-reactive protein, IL-6, or TNF-α levels,^18^^,^^91^ with only trends toward lower serum concentrations. Future studies should combine the assessment of adhesion molecules, inflammatory markers, and parameters of endothelial function such as PWV and AIX.

Regarding hunger and satiety, analysis of hunger- and satiety-related hormones in the included studies revealed that the postprandial concentrations of anorexic peptides and orexigenic hormones were higher and lower, respectively, following meals with non-oil-seed pulses than following control meals. These effects on hunger- and satiety-related hormones indicate a reduced postprandial hunger and an increased satiety following meals containing legumes than following control meals. Despite the unequivocal results regarding hunger- and satiety-related hormones, the results for subjective hunger, appetite, satiety, and fullness sensations measured via visual analog scales are more heterogenous, although some of the studies^41^^,^^47^^,^^50^^,^^55^^,^^56^ reported increased satiety or fullness ratings following pulse meals, while hunger and appetite ratings were decreased. However, most of the studies did not find significant changes in hunger or satiety sensations between intervention and control meals.^40^^,^^42^^,^^43^^,^^46^^,^^49^^,^^53^^,^^57^^,^^59^^,^^60^^,^^62^^,^^63^^,^^67^^,^^71^ The reported effects regarding the postprandial concentrations of hormones that regulate hunger and satiety after the consumption of pulse meals were expected, because the protein present in non-oil-seed pulses can increase the release of CCK, PYY, and GLP-1.^91^ In addition to the changes in anorexic and orexigenic peptides in the circulation, the higher satiation and lower hunger sensations following pulse meals observed in some of the studies could be explained by the content of dietary fiber in the meals, resulting in slower gastric emptying and leading to prolonged mechanical stimuli for satiety regulation. Furthermore, slower gastric emptying and increased viscosity in the small intestine lead to slower absorption of macronutrients like protein, carbohydrates, and fats into the blood stream and thus levels of satiety-associated hormones are secreted over a longer period of time.^92^ However, reduced *ad libitum* food intake in a second meal following non-oil-seed-based meals compared with the control was observed in only 2 of 9 studies. This contrasts with chronic studies suggesting that inclusion of pulses in a diet is a beneficial weight-loss strategy.^30^ In the acute setting, *ad libitum* food intake was lower, albeit not significantly, following meals containing non-oil-seed pulses than following control meals. Therefore, the integration of non-oil-seed pulses in meals might lower daily food intake in the long run and therefore decrease body weight over time.

To our knowledge, this is the first systematic review to systematically investigate the acute effects of different non-oil-seed pulses on parameters of cardiometabolic health as well as on hunger and satiety in healthy adults and individuals with increased cardiovascular disease risk. This review facilitates the development of nutritional recommendations to reduce postprandial glycemia and insulinemia as well as to increase and prolong satiety after meal intake.

### Challenges, Limitations, and Perspectives

Despite promising findings, this review highlights substantial heterogeneity across the study designs, populations, and intervention types. Many of the included intervention studies lacked a standardization in macronutrient composition or administered diverse legume preparations, thereby limiting comparability. Consequently, no meta-analysis was conducted, and no definitive intake thresholds of pulses could be established for eliciting effects on postprandial parameters such as glucose concentrations or satiety. Moreover, only a limited number of studies examined vascular, inflammatory, or satiety-related end points in depth. Future research should focus on standardized, isoenergetic interventions and incorporate a broad range of biomarkers and mechanistic outcomes—particularly in populations with metabolic disorders. Additionally, examining processing methods that preserve bioactive compounds will be critical for better understanding of how processing of legumes influences postprandial responses. Beyond their potential role as an alternative carbohydrate source, pulses are also rich in protein (Table 1). The Planetary Health Diet, which aims to align human and environmental health, emphasizes pulses as an important plant protein source for addressing the challenges posed by global population growth and climate change.^4^ Furthermore, various dietary guidelines classify legumes as protein sources rather than as carbohydrate-rich foods or vegetables.^13^^,^^14^ Accordingly, future research should not only compare the effects of non-oil-seed pulses on postprandial metabolism relative to starchy foods but also evaluate their impact as a plant-based protein sources in comparison with other protein sources.

## CONCLUSION

Taken together, the results of this review have demonstrated that addition of non-oil-seed pulses to meals, or exchange of non-oil-seed pulses for other carbohydrate sources, attenuates postprandial glycemia and insulinemia and might reduce hunger and increase satiety. Only a few studies have investigated additional postprandial parameters and systems, such as lipid and protein metabolism, endothelial function, and inflammation. Therefore, further research is necessary to fully understand the role of non-oil-seed pulses in the regulation of postprandial metabolism and their role as a plant-based protein source.

## Supplementary Material

nuaf190_Supplementary_Data
