# Supplementary material for: Acute Effects of Non-Oil-Seed Pulses on Parameters of Cardiometabolic Health: A Systematic Review of Human Intervention Studies
Source: Nutr Rev. 2025 Nov 29;84(9):1848–61. doi: 10.1093/nutrit/nuaf190 (PMC13563096; doi:10.1093/nutrit/nuaf190)
Supplement: Supplementary file 1 [file nuaf190_supplementary_data.zip › Schiemann et al. Supplementary Table.docx]

Supplementary table 1. Included studies

| **Reference** | **Subjects** | **Study design** | **Postprandial period (time points)** | **Intervention** | **Relevant outcomes** | **Results intervention vs. control** |
| --- | --- | --- | --- | --- | --- | --- |
| Akhtar et al. (2019) ^56^ | 24 healthy subjects, 22.5 years old | Randomized crossover study | 120 min (0, 15, 30, 45, 60, 90, and 120 min) | A. Chapatti enriched with kidney bean (10 g) and gram (15 g) flour   1030 kJ, 1.1 g fat, 8.9 g protein, 50 g carbohydrate, and 5.3 g fiber   B. Wheat flour-based chapatti   959 kJ, 0.8 g fat, 5 g protein, 50 g carbohydrate, and 3.6 g fiber | Glucose and insulin | AUCs of glucose and insulin ↓* |
| Alshaalan et al. (2024) ^70^ | 15 healthy men and women, 33 years old | Randomized crossover study | 180 min (0, 15, 30, 60, 90, 120, and 180 min) | A. Hummus meal (RCC - ruptured chickpea cells, 76.5 g dry-milled flour)   510 kcal, 30.1 g fat, and 26.8 g starch   B. Hummus meal (ICC - intact chickpea cells, 157 g canned chickpeas)   460 kcal, 29.5 g fat, and 26.8 g starch | Glucose, insulin, GIP, and GLP-1 | iAUC of insulin ↓* after ICC meal; insulin ↓* at 30 and 120 min after ICC meal; GIP ↓* at 15, 30, and 60 min after ICC meal; no effects on GLP-1 and glucose |

Supplementary table 1. Continued

| **Reference** | **Subjects** | **Study design** | **Postprandial period (time points)** | **Intervention** | **Relevant outcomes** | **Results intervention vs. control** |
| --- | --- | --- | --- | --- | --- | --- |
| Anderson et al. 2014 ^64^ | 17 healthy males, 22.9 years old | Randomized crossover study, standardized breakfast before intervention | 120 min (0, 15, 30, 45, 60, 90, and 120 min) | A. Whole navy beans (188 g)   980 kJ, 1.5 g fat, 17.5 g protein, 55 g carbohydrate, and 16.3 g fiber   B. Navy bean powder (62.5 g)   1040 kJ, 1.7 g fat, 20.5 g protein, 60.1 g carbohydrate, and 21.4 g fiber   C. Pureed navy beans (188 g)   980 kJ, 1.5 g fat, 17.5 g protein, 55 g carbohydrate, and 16.3 g fiber   D. Whole wheat flour   817 kJ, 0.7 g fat, 9.6 g protein, 46.6 g carbohydrate, and 7.9 g fiber  Standardized for available carbohydrates (38.7 g) | Glucose | Peak glucose concentration ↓* (for all navy bean arms); no significant effects on AUC |

Supplementary table 1. Continued

| **Reference** | **Subjects** | **Study design** | **Postprandial period (time points)** | **Intervention** | **Relevant outcomes** | **Results intervention vs. control** |
| --- | --- | --- | --- | --- | --- | --- |
| Anderson et al. (2014) ^64^ | 12 healthy males, 22.2 years old | Randomized crossover study, standardized breakfast before intervention | 120 min (0, 15, 30, 45, 60, 90, and 120 min) | A. Whole lentils (183.8 g)   944 kJ, 0.9 g fat, 16.5 g protein, 50 g carbohydrate, and 11.3 g fiber   B. Lentil powder (47.7 g)   924 kJ, 0.8 g fat, 15.5 g protein, 49 g carbohydrate, and 10.3 g fiber   C. Pureed lentils (183.8 g)   944 kJ, 0.9 g fat, 16.5 g protein, 50 g carbohydrate, and 11.3 g fiber   D. Whole wheat flour   842 kJ, 0.9 g fat, 10.5 g protein, 46.6 g carbohydrate, and 7.9 g fiber | Glucose | Mean glucose ↓* (for all lentil arms) |

Supplementary table 1. Continued

| **Reference** | **Subjects** | **Study design** | **Postprandial period (time points)** | **Intervention** | **Relevant outcomes** | **Results intervention vs. control** |
| --- | --- | --- | --- | --- | --- | --- |
| Continued Anderson et al. (2014) ^64^ | 12 healthy males, 23.6 years old | Randomized crossover study, standardized breakfast before intervention | 120 min (0, 15, 30, 45, 60, 90, and 120 min) | A. Whole chickpeas (148.8 g)   1006 kJ, 3.6 g fat, 14.3 g protein, 49.2 g carbohydrate, and 10.5 g fiber   B. Chickpea powder (49.7 g)   1027 kJ, 3.5 g fat, 15.6 g protein, 48.3 g carbohydrate, and 9.6 g fiber   C. Pureed chickpeas (148.8 g)   1006 kJ, 3.6 g fat, 14.3 g protein, 49.2 g carbohydrate, and 10.5 g fiber   D. Whole wheat flour   842 kJ, 0.9 g fat, 10.5 g protein, 46.6 g carbohydrate, and 7.9 g fiber | Glucose | Mean glucose ↓* (for all chickpea arms) |

Supplementary table 1. Continued

| **Reference** | **Subjects** | **Study design** | **Postprandial period (time points)** | **Intervention** | **Relevant outcomes** | **Results intervention vs. control** |
| --- | --- | --- | --- | --- | --- | --- |
| Augustin et al. (2016) ^35^ | 10 overweight men and women, 53 years old | Randomized crossover study | 120 min (0, 15, 30, 45, 60, 90, and 120 min) | A. White bread   25 g available carbohydrates   B. Hummus (28 g)   2.7 g available carbohydrates   C. Hummus (112 g)   10.8 g available carbohydrates   D. Hummus (259 g)   25 g available carbohydrates   A. White bread   50 g available carbohydrates   B. White bread plus hummus (28 g)   52.7 g available carbohydrates   C. White bread plus hummus (56 g)   55.4 g available carbohydrates   D. White bread plus hummus (112 g)   60.8 g available carbohydrates | Glucose and insulin | AUC of glucose ↓* (for all hummus meals); AUC of insulin ↓* (for 28 and 112 g hummus)  No significant differences between the AUCs of glucose and insulin (for white bread and different amounts of hummus) |

Supplementary table 1. Continued

| **Reference** | **Subjects** | **Study design** | **Postprandial period (time points)** | **Intervention** | **Relevant outcomes** | **Results intervention vs. control** |
| --- | --- | --- | --- | --- | --- | --- |
| Bajka et al. (2023) ^38^ | 20 healthy subjects, 27.9 years old | Randomized crossover study | 240 min (0, 15, 30, 45, 60, 90, 120, 180, and 240 min) | A. White bread enriched with chickpea flour (30%)   1502 kJ, 5.6 g fat, 7.7 g protein, 48.3 g carbohydrate, and 6.2 g fiber   B. White bread enriched with chickpea flour (60%)   1823 kJ, 8.8 g fat, 27.2 g protein, and 48.2 g carbohydrate   C. White bread control   1302 kJ, 3.3 g fat, 12.9 g protein, 48.1 g carbohydrate | Glucose, insulin, C-peptide, GLP-1, serum total amino acids, PYY, satiety, and *ad libitum* food intake | AUCs of amino acids, GLP-1, and PYY ↑ (for 30% enrichment) and ↑* (for 60% enrichment); AUC of glucose ↓* (for both treatments); AUCs of insulin and C-peptide ↓ (for 30% enrichment) and ↓* (for 60% enrichment); satiety ↑ (for 60% enrichment); *ad libitum* food intake ↓ (for 60% enrichment) |
| Boers et al. (2017) ^42^ | 38 healthy subjects, 37 years old | Randomized balanced-order incomplete block design | 180 min (0, 30, 60, 90, 120, 150, and 180 min) | A. Whole wheat flour bread enriched with chickpea flour (15 g)   57 g available carbohydrates and 13 g fiber   B. Whole wheat flour bread   61 g available carbohydrates and 11 g fiber | Glucose | iAUC of glucose ↑ |

Supplementary table 1. Continued

| **Reference** | **Subjects** | **Study design** | **Postprandial period (time points)** | **Intervention** | **Relevant outcomes** | **Results intervention vs. control** |
| --- | --- | --- | --- | --- | --- | --- |
| Bourdon et al. (2001) ^68^ | 10 healthy men, 38.1 years old | Randomized crossover study | 360 min (0, 30, 45, 60, 120, 180, 240, 300, and 360 min) | A. Bean flakes meal (60 g)   3060 kJ, 18.7 g fat, 34.7 g protein, and 106 g carbohydrate   B. Instant rice and nonfat milk powder   3185 kJ, 18.6 g fat, 34.2 g protein, and 114.1 g carbohydrate | Glucose, insulin, TAGs, HDL, LDL, and CCK | AUCs of glucose and insulin ↓; AUC of TAGs ↑; AUC of CCK ↑*; no effect on LDL and HDL |
| Chan et al. (2019) ^51^ | 42 healthy men, 23.4 years old | Randomized crossover study, standardized breakfast 4 h before intervention | 120 min (0, 15, 30, 45, 60, 90, and 120 min) | A. Pasta enriched with split faba bean flour (25%)   368 kcal, 1.5 g fat, 18.6 g protein, 71.7 g carbohydrate, and 7.2 g fiber   B. Durum wheat pasta   364 kcal, 1.6 g fat, 15.1 g protein, 75.8 g carbohydrate, and 5.9 g fiber | Glucose, insulin, C-peptide, GLP-1, appetite, and PYY | Mean glucose and C-peptide ↓; mean insulin and GLP-1 ↑; appetite ↑; PYY ↑* |

Supplementary table 1. Continued

| **Reference** | **Subjects** | **Study design** | **Postprandial period (time points)** | **Intervention** | **Relevant outcomes** | **Results intervention vs. control** |
| --- | --- | --- | --- | --- | --- | --- |
| Chamoun et al. (2024) ^71^ | 24 healthy men and women, 26.9 years old | Randomized crossover study | 120 min (0, 15, 30, 45, 60, 90, and 120 min) | A. Muffin (wheat control)   384 kcal, 9.9 g fat, 9.5 g protein 58.3 g carbohydrate, and 4.2 g fiber   B. Muffin enriched with green lentils   505 kcal, 10.4 g fat, 20 g protein, 71.5 g carbohydrate, and 15.1 g fiber   C. Muffin enriched with red lentils   458 kcal, 10.3 g fat, 16.8 g protein, 65.2 g carbohydrate, and 9.8 g fiber  Standardized to provide 25 g available carbohydrates from lentils or starch-rich control | Glucose and insulin | AUC of glucose ↓* (for red lentil muffin compared with control); AUC of insulin ↓* (for green lentil muffin compared with control) |

Supplementary table 1. Continued

| **Reference** | **Subjects** | **Study design** | **Postprandial period (time points)** | **Intervention** | **Relevant outcomes** | **Results intervention vs. control** |
| --- | --- | --- | --- | --- | --- | --- |
| Continued Chamoun et al. (2024) ^71^ | 24 healthy men and women, 26.6 years old  20 healthy men and women, 23.1 years old |  |  | A. Rice with chili   194 kcal, 2.2 g fat, 4.8 g protein, 46.8 g carbohydrate, and 5 g fiber   B. Green lentils with chili   314 kcal, 3.3 g fat, 46.8 g carbohydrate, and 16.9 g fiber   C. Red lentils with chili   287 kcal, 3 g fat, 14.7 g protein, 43.5 g carbohydrate, and 13.1 g fiber  Standardized to provide 25 g available carbohydrates from lentils or starch-rich control   A. Soup with potatoes   190 kcal, 1.3 g fat, 5.6 g protein, 36.7 g carbohydrate, and 5.8 g fiber   B. Soup with green lentils   276 kcal, 1.7 g fat, 15.7 g protein, 43.1 g carbohydrate, and 13.9 g fiber   C. Soup with red lentils   245 kcal, 1.6 g fat, 13.3 g protein, 38.3 g carbohydrate, and 9.7 g fiber  Standardized to provide 25 g available carbohydrates from lentils or starch-rich control |  | AUCs of glucose and insulin ↓* (for both lentil meals compared with control)  AUCs of glucose and insulin ↓* (for both lentil meals compared with control) |

Supplementary table 1. Continued

| **Reference** | **Subjects** | **Study design** | **Postprandial period (time points)** | **Intervention** | **Relevant outcomes** | **Results intervention vs. control** |
| --- | --- | --- | --- | --- | --- | --- |
| Clark et al. (2020) ^67^ | 8 healthy men and women, 37 years old | Randomized crossover pilot study | 360 min (0, 60, 120, and 360 min) | A. White rice (103.5 g cooked weight) B. Navy beans (187.5 g cooked weight) C. Pinto beans (182.7 g cooked weight) D. Red kidney beans (177.4 g cooked weight) E. Black beans (181.4 g cooked weight) | Glucose, TAGs, TC, LDL, HDL, PWV, and AIX | No significant effects on glucose, TAGs, TC, HDL, and AIX; LDL ↓* at 6 h after black beans compared with white rice; PWV ↓* at 2 h after kidney beans and pinto beans compared with navy beans and ↓* at 6 h after black beans compared with pinto beans |
| Fahmi et al. (2022) ^45^ | 24 healthy subjects, 24.5 years old | Randomized crossover study | 120 min (0, 15, 30, 45, 60, 90, and 120 min) | A. Wheat bread enriched with split yellow pea flour (20%)   269 kcal, 2.6 g fat, 12.1 g protein, 50 g carbohydrate, and 0.7 g fiber   B. Wheat bread   256 kcal, 2.2 g fat, 9.3 g protein, 50 g carbohydrate, and 1 g fiber | Glucose, insulin, hunger, and fullness | AUC of glucose ↓;  AUC of insulin ↑;  AUC of hunger ↓*; AUC of fullness ↑* |

Supplementary table 1. Continued

| **Reference** | **Subjects** | **Study design** | **Postprandial period (time points)** | **Intervention** | **Relevant outcomes** | **Results intervention vs. control** |
| --- | --- | --- | --- | --- | --- | --- |
| Hafiz et al. (2022) ^39^ | 13 healthy subjects, 28.7 years old | Randomized crossover study | 180 min (0, 30, 60, 90, 120, 150, and 180 min) | A. Whole chickpeas (250 g)   1461 kJ, 8 g fat, 19.3 g protein, 50 g carbohydrate, and 15.3 g fiber   B. Chickpea puree (250 g)   1448 kJ, 8 g fat, 19.3 g protein, 50 g carbohydrate, and 15.3 g fiber   C. Chickpea pasta (217 g)   1497 kJ, 8 g fat, 21.3 g protein, 50 g carbohydrate, and 12.4 g fiber   D. Mashed potato (425 g)   1242 kJ, 8 g fat, 6.2 g protein, 50 g carbohydrate, and 4.7 g fiber | Glucose, GLP-1, C-peptide, hunger, and fullness | AUCs of glucose, hunger, GLP-1, and C-peptide ↓* (for all chickpea treatments); AUC of fullness ↑* (for all chickpea treatments) |
| Hall et al.  (2005) ^47^ | 11 healthy subjects, 31.6 years old | Randomized crossover study | 120 min (15, 30, 45, 60, 90, and 120 min) | A. Australian sweet lupin bread (10%)   1338 kJ, 7.3 g fat, 12.8 g protein, 50 g carbohydrate, and 4.9 g fiber   B. White bread   1243 kJ, 6.4 g fat, 9.2 g protein, 50 g carbohydrate, 2.8 g fiber | Glucose, insulin, satiety, and *ad libitum* food intake | Incremental glucose concentration ↓; incremental insulin concentration, satiety, and *ad libitum* food intake ↑ |

Supplementary table 1. Continued

| **Reference** | **Subjects** | **Study design** | **Postprandial period (time points)** | **Intervention** | **Relevant outcomes** | **Results intervention vs. control** |
| --- | --- | --- | --- | --- | --- | --- |
| Johnson et al. (2004) ^40^ | 11 healthy subjects, 32 years old | Randomized crossover study | 120 min (0, 15, 30, 45, 60, 90, and 120 min) | A. Chickpea bread (24.3%)   1334 kJ, 7 g fat, 11 g protein, 50 g carbohydrate, and 5 g fiber   B. Extruded chickpea bread (24.3%)   1342 kJ, 7 g fat, 11 g protein, 50 g carbohydrate, and 6 g fat   C. White bread   1251 kJ, 6 g fat, 9 g protein, 50 g carbohydrate, and 3 g fiber | Glucose, insulin, satiety, and *ad libitum* food intake | AUC of glucose ↓ (for both chickpea treatments); AUC of insulin ↑* (for chickpea bread) and ↑ (for extruded chickpea bread); no significant effect on satiety (for chickpea bread) and ↑ (for extruded chickpea bread); no significant effect on *ad libitum* food intake |

Supplementary table 1. Continued

| **Reference** | **Subjects** | **Study design** | **Postprandial period (time points)** | **Intervention** | **Relevant outcomes** | **Results intervention vs. control** |
| --- | --- | --- | --- | --- | --- | --- |
| Johnston et al. (2020) ^41^ | 26 healthy subjects, 24.7 years old | Randomized crossover study | 120 min (0, 15, 30, 45, 60, 90, and 120 min) | A. Corn snack with 40% whole yellow pea flour 198 kcal B. Corn snack with 40% split yellow pea flour 204 kcal C. Corn snack with 40% green lentil flour 198 kcal D. Corn snack with 40% chickpea flour 202 kcal E. Corn snack with 40% pinto bean flour 199 kcal F. Corn snack   209 kcal | Glucose, insulin, appetite, and *ad libitum* food intake | AUC of glucose ↓* (for pinto bean and chickpea snacks) ↓ (for split yellow pea and green lentil snacks), and ↑ (for whole yellow pea snack) and insulin ↓* (for pinto bean snack), ↓ (for green lentil, whole yellow pea flour and chickpea snacks), and ↑ (for split yellow pea snack); AUC of appetite and *ad libitum* food intake ↓ (for whole yellow pea snack) and ↑ (for split yellow pea, green lentil, chickpea, and pinto bean snacks) |

Supplementary table 1. Continued

| **Reference** | **Subjects** | **Study design** | **Postprandial period (time points)** | **Intervention** | **Relevant outcomes** | **Results intervention vs. control** |
| --- | --- | --- | --- | --- | --- | --- |
| Keogh et al. (2011) ^48^ | 20 healthy subjects, 29.4 years old | Randomized crossover study | 120 min (0, 15, 30, 45, 60, 90, and 120 min) | A. Lupin bread (n.i.)   1266 kJ, 10.1 g fat, 14.1 g protein, 37.3 g carbohydrate, and 8.1 g fiber   B. White bread   1339 kJ, 9.1 g fat, 7.1 g protein, 53.7 g carbohydrate, and 2.3 g fiber | Glucose, insulin, fullness, and *ad libitum* food intake | AUCs of glucose and insulin ↓*; fullness ↑*; *ad libitum* food intake ↓ |
| Lee et al.  (2006) ^49^ | 17 healthy subjects, 61 years old | Non-randomized crossover study | 180 min (0, 15, 30, 45, 60, 90, 120, 150, and 180 min) | A. Lupin bread (40%)   1400 kJ, 5.7 g fat, 25 g protein, 40 g carbohydrate, and 15 g fiber   B. White bread   1400 kJ, 5.7 g fat, 12 g protein, 57 g carbohydrate, and 3.4 g fiber | Glucose, insulin, and ghrelin | AUCs of glucose, ghrelin, and insulin ↓* |

Supplementary table 1. Continued

| **Reference** | **Subjects** | **Study design** | **Postprandial period (time points)** | **Intervention** | **Relevant outcomes** | **Results intervention vs. control** |
| --- | --- | --- | --- | --- | --- | --- |
| Marinangeli et al. (2009) ^46^ | 22 healthy men and women, 22–67 years old | Randomized crossover study | 150 min (0, 30, 60, 120, and 150 min) | A. Whole yellow pea flour banana bread   1544 kJ, 15.2 g fat, 9.3 g protein, 52 g carbohydrate, and 8.1 g fiber   B. Whole wheat flour banana bread   1523 kJ, 16.1 g fat, 7.8 g protein, 51.7 g carbohydrate, and 7.1 g fiber   C. Whole yellow pea flour biscotti   1503 kJ, 13.3 g fat, 12.2 g protein, 51.7 g carbohydrate, and 10.1 g fiber   D. Whole wheat flour biscotti   1458 kJ, 13.3 g fat, 9.5 g protein, and 53.2 g carbohydrate   E. Whole yellow pea flour pasta   1134 kJ, 1.6 g fat, 7.4 g protein, 51.1 g carbohydrate, and 8.1 g fiber   F. Whole wheat flour pasta   1.2 g fat, 9.9 g protein, 51.1 g carbohydrate, and 6.6 g fiber | Glucose | AUC of glucose ↓* (for whole yellow pea flour banana bread and whole yellow pea flour biscotti) and ↓ (for yellow pea flour pasta) |
|  |  |  |  |  |  |  |

Supplementary table 1. Continued

| **Reference** | **Subjects** | **Study design** | **Postprandial period (time points)** | **Intervention** | **Relevant outcomes** | **Results intervention vs. control** |
| --- | --- | --- | --- | --- | --- | --- |
| Mollard et al. (2011) ^58^ | 24 healthy men, 24.3 years old | Randomized crossover study, standardized breakfast 4 h before intervention | 260 min (0, 20, 40, 60, 80, 110, 140, 200, and 260 min) | A. Chickpeas   Per 100 g: 324.5 kJ, 1.7 g fat, 3.5 g protein, 12 g carbohydrate, and 2.1 g fiber   B. Lentils   Per 100 g: 323.2 kJ, 1.3 g fat, 3.9 g protein, 12.6 g carbohydrate, and 2.5 g fiber   C. Navy beans   Per 100 g: 324.1 kJ, 1.4 g fat, 4.2 g protein, 12 g carbohydrate, and 3.2 g fiber   D. Yellow peas   Per 100 g: 327.4 kJ, 1.2 g fat, 3.9 g protein, 12.9 g carbohydrate, and 2 g fiber   E. Pasta and sauce   Per 100 g: 322 kJ, 1.3 g fat, 2.6 g protein, 13.7 g carbohydrate, and 0.9 g fiber  Subjects ate until they were comfortably full | Glucose and *ad libitum* food intake | AUC of glucose ↓* (for all treatments except yellow peas) and ↓ (for yellow peas); appetite and *ad libitum* food intake ↓ (for all treatments) |

Supplementary table 1. Continued

| **Reference** | **Subjects** | **Study design** | **Postprandial period (time points)** | **Intervention** | **Relevant outcomes** | **Results intervention vs. control** |
| --- | --- | --- | --- | --- | --- | --- |
| Mollard et al. (2011) ^57^ | 20 healthy men, 21.3 years old | Randomized crossover study | 260 min (0, 20, 40, 60, 80, 110, 140, 200, and 260 min) | A. Chickpeas (223 g) and macaroni   603 kcal, 12.5 g fat, 26.1 g protein, 98.7 g carbohydrate, and 18.3 g fiber   B. Lentils (333 g) and macaroni   597 kcal, 8.7 g fat, 29.1 g protein, 103.2 g carbohydrate, and 18.7 g fiber   C. Yellow peas (376 g) and macaroni   596 kcal, 8.5 g fat, 28.3 g protein, 103.5 g carbohydrate, and 14.1 g fiber   D. Macaroni and cheese   604 kcal, 12.6 g fat, 100.4 g carbohydrate, and 2.8 g fiber | Glucose, appetite, and *ad libitum* food intake | AUC of blood glucose ↓ (for all treatments); appetite ↓ (for all treatments); *ad libitum* food intake ↓* (for lentil and yellow pea meals) and ↓ (for chickpea meal) |
| Mollard et al. (2014) ^65^ | 15 healthy men, 21.5 years old | Randomized crossover study | 135 min (0, 15, 30, 45, 75, and 135 min) | A. Yellow peas with tomato sauce   322 kcal, 6 g fat, 18 g protein, 49 g carbohydrate, and 10 g fiber   B. Noodles with tomato sauce   324 kcal, 4 g fat, 10 g protein, 62 g carbohydrate, and 2 g fiber | Glucose, appetite, and *ad libitum* food intake | AUC of glucose ↓*; appetite and *ad libitum* food intake ↓ |

Supplementary table 1. Continued

| **Reference** | **Subjects** | **Study design** | **Postprandial period (time points)** | **Intervention** | **Relevant outcomes** | **Results intervention vs. control** |
| --- | --- | --- | --- | --- | --- | --- |
| Naseer et al. (2019) ^54^ | 12 healthy females, 18–30 years old | Randomized crossover study | 120 min (0, 15, 30, 45, 60, 90, and 120 min) | A. Lentil-based muffin (20%)   350 kcal, 1.4 g fat, 22.5 g protein, 60.3 g carbohydrate, and 3.5 g fiber   B. Mung bean-based muffin (20%)   349 kcal, 1.1 g fat, 24.8 g protein, 56.1 g carbohydrate, and 3.6 g fiber   C. Lentil (10%)- and mung bean (10%)-based muffin   345 kcal, 1.3 g fat, 23.7 g protein, 58.2 g carbohydrate, and 3.5 g fiber   D. Control   350 kcal, 1.4 g fat, 10.8 g protein, 75 g carbohydrate, and 0.8 g fiber | Glucose, appetite, and *ad libitum* food intake | AUC of glucose ↓* (for all treatments); appetite ↓* (for all treatments); *ad libitum* food intake ↓ |
| Nestel et al. (2004) ^59^ | 19 healthy subjects, 61.5 years old | Crossover study | Glucose: 180 min (0, 30, 60, 120, and 180 min)  Insulin and HOMA: 120 min (120 min) | A. Smashed chickpeas (200 g) B. White bread C. Wheat cream   Standardized for available carbohydrates (50 g), 12 g protein, <2 g fat, and 10 g fiber | Glucose, insulin, and HOMA | Glucose ↓*; insulin and HOMA at 120 min ↓* |

Supplementary table 1. Continued

| **Reference** | **Subjects** | **Study design** | **Postprandial period (time points)** | **Intervention** | **Relevant outcomes** | **Results intervention vs. control** |
| --- | --- | --- | --- | --- | --- | --- |
| Nestel et al. (2004) ^59^ (same paper, different study) | 10 healthy subjects, n.i. | Crossover study | 420 min (0, 60, 120, 180, 300, and 420 min) | A. Cooked chickpeas (100 g) and cream, cheese, butter, and whole milk B. Bread made of chickpea flour and cream, cheese, butter, and whole milk C. White bread, rice bubbles, and cream, cheese, butter, and whole milk D. Shredded wheat cereal and whole grain bread and cream, cheese, butter, and whole milk   Standardized for 65 g starch and 56 g fat from dairy products; meals contained 11–14 g protein, 12–14 g fiber, and 2–7 g additional fat | TAGs, LDL, and HDL | No significant effects on TAGs, LDL, and HDL |
| Olmedilla-Alonso et al. (2013) ^32^ | 12 type 2 diabetics, 66.4 years old | Randomized crossover study | 360 min (0, 30, 60, 90, 120, 180, 240, and 360 min) | A. Almonga beans (275 g)   57.8 g carbohydrate   B. Curruquilla beans (275 g)   52.3 g carbohydrate   C. White bread (114 g)   59.3 g carbohydrate  Each meal was served with 20 g olive oil | Glucose, insulin, and TAGs | AUCs of glucose and insulin ↓* (for both treatments); AUC of TAGs ↓* (for almonga beans) and ↓ (for curruquilla beans) |

Supplementary table 1. Continued

| **Reference** | **Subjects** | **Study design** | **Postprandial period (time points)** | **Intervention** | **Relevant outcomes** | **Results intervention vs. control** |
| --- | --- | --- | --- | --- | --- | --- |
| Papakonstantinou et al. (2022) ^60^ | 12 healthy subjects, 23.3 years old | Randomized crossover study | 120 min (15, 30, 45, 60, 90, and 120 min) | A. Legume meal with lupin (378 g)   284 kcal, 7.2 g fat, 23.5 g protein, 41.7 g carbohydrate, and 18.8 g fiber   B. Trahana meal   270 kcal, 2.4 g fat, 8.3 g protein, 48.7 g carbohydrate, and 10.5 g fiber   C. Halva meal   308 kcal, 9.3 g fat, 6.4 g protein, 53 g carbohydrate, and 5.1 g fiber | Glucose and appetite | AUC of glucose ↓* (compared with the halva meal) and ↓ (compared with the trahana meal); no effects on appetite |

Supplementary table 1. Continued

| **Reference** | **Subjects** | **Study design** | **Postprandial period (time points)** | **Intervention** | **Relevant outcomes** | **Results intervention vs. control** |
| --- | --- | --- | --- | --- | --- | --- |
| Ramdath et al. (2017) ^66^ | 10 healthy subjects, 45.1 years old | Randomized crossover study | 120 min (15, 30, 45, 60, 90, and 120 min) | A. Asterix lentils   866 kJ, 0.4 g fat, 14.2 g protein, 36.7 g carbohydrate, and 11.7 g fiber   B. Imigreen lentils   872 kJ, 0.6 g fat, 14 g protein, 36.7 g carbohydrate, and 11.7 g fiber   C. Impower lentils   865 kJ, 0.7 g fat, 13.5 g protein, 36.7 g carbohydrate, and 11.7 g fiber   D. Improve lentils   1016 kJ, 0.8 g fat, 16.7 g protein, 42.4 g carbohydrate, and 17.4 g fiber   E. Redberry lentils   1054 kJ, 1 g fat, 17.9 g protein, 42.8 g carbohydrate, and 17.8 g fiber   F. Greenland lentils   965 kJ, 0.6 g fat, 15.6 g protein, 40.8 g carbohydrate, and 15.8 g fiber   G. Redcliff lentils   976 kJ, 0.8 g fat, 15.5 g protein, 41 g carbohydrate, and 16 g fiber | Glucose | AUC of glucose ↓* (for all lentils compared with control) |

Supplementary table 1. Continued

| **Reference** | **Subjects** | **Study design** | **Postprandial period (time points)** | **Intervention** | **Relevant outcomes** | **Results intervention vs. control** |
| --- | --- | --- | --- | --- | --- | --- |
| Continued - Ramdath et al. (2017) ^66^ |  |  |  | H. Redbow lentils   1047 kJ, 0.4 g fat, 17.6 g protein, 43.8 g carbohydrate, and 18.9 g fiber   I. White bread   543 kJ, 0.3 g fat, 5.4 g protein, 26 g carbohydrate, and 1.3 g fiber  Standardized for available carbohydrates (25 g) |  |  |
| Reister et al. (2020) ^69^ | 39 healthy subjects, 26 years old | Randomized crossover study, standardized breakfast and lunch before the snack intervention | 180 min (continuous glucose monitoring) | A. Hummus (57 g) snack with pretzels   240 kcal, 11 g fat, 6 g protein, 31 g carbohydrate, and 4 g fiber   B. Granola bar   240 kcal, 9 g fat, 4 g protein, 38 g carbohydrate, and 2 g fiber | Glucose, appetite, and fullness | AUC of glucose ↓*; appetite ↓; fullness ↑ |

Supplementary table 1. Continued

| **Reference** | **Subjects** | **Study design** | **Postprandial period (time points)** | **Intervention** | **Relevant outcomes** | **Results intervention vs. control** |
| --- | --- | --- | --- | --- | --- | --- |
| Reverri et al. (2015) ^36^ | 12 men and women with metabolic syndrome, 49 years old | Randomized crossover pilot study | 300 min (0, 60, 120, 180, 240, and 300 min) | A. Black bean meal   919 kcal, 25 g fat, 42 g protein, 129 g carbohydrate, and 18 g fiber   B. Fiber-matched meal   933 kcal, 25 g fat, 39 g protein, 135 g carbohydrate, and 17 g fiber   C. Antioxidant capacity-matched meal   923 kcal, 25 g fat, 39 g protein, 133 g carbohydrate, and 4 g fiber | Glucose, insulin, TAGs, IL-6, sICAM1, sVCAM1, total ORAC, and oxidized LDL | No significant effects on TAG, glucose, oxidized LDL, and IL-6 concentrations; insulin concentration ↓* (for black bean meal compared with fiber- and antioxidant capacity-matched meals); sICAM1 and sVCAM1 ↑* and ORAC ↓* (for black bean meal compared with antioxidant capacity-matched meal) |

Supplementary table 1. Continued

| **Reference** | **Subjects** | **Study design** | **Postprandial period (time points)** | **Intervention** | **Relevant outcomes** | **Results intervention vs. control** |
| --- | --- | --- | --- | --- | --- | --- |
| Rossukon et al. (2024) ^55^ | 12 healthy subjects, 26.8 years old | Randomized crossover study | 120 min (0, 15, 30, 45, 60, 90, and 120 min) | A. Wheat cracker   458 kcal, 12.6 g fat, 7 g protein, 79 g carbohydrate, and 2.4 g fiber   B. Cashew nut cracker   469 kcal, 15.4 g fat, 8.8 g protein, 73.7 g carbohydrate, and 3.1 g fiber   C. Almond cracker   482 kcal, 17.7 g fat, 9.8 g protein, 70.6 g carbohydrate, and 3.2 g fiber   D. White kidney bean cracker 446 kcal, 11.3 g fat, 91.1 g protein, 77.3 g carbohydrate, and 1.8 g fiber   Standardized for available carbohydrates (50 g), in all crackers 30% of wheat flour was replaced by the investigated flour | Glucose, hunger, and satiety | No significant effects on glucose, hunger, and satiety |

Supplementary table 1. Continued

| **Reference** | **Subjects** | **Study design** | **Postprandial period (time points)** | **Intervention** | **Relevant outcomes** | **Results intervention vs. control** |
| --- | --- | --- | --- | --- | --- | --- |
| Schäfer et al. (2003) ^33^ | 9 type 2 diabetics, 61 years old | Randomized crossover study, breakfast and snack at 4.5/2.5 h before intervention | 180 min (0, 30, 60, 90, 120, 150, and 180 min) | A. Dried peas (90 g)   2256 kJ, 27.4 g fat, 30.6 g protein, 40.6 g carbohydrate, and 18.8 g fiber   B. Mixed meal (60 g peas)   2134 kJ, 27.1 g fat, 25.3 g protein, 40 g carbohydrate, and 15.6 g fiber   C. Potatoes   2152 kJ, 26.1 g fat, 29.6 g protein, 41.6 g carbohydrate, and 10.9 g fiber | Glucose and insulin | AUC of glucose ↓* (for pea and mixed meals); AUC of insulin ↓* (for pea and mixed meals) |
| Schopen et al. (2017) ^50^ | 12 healthy subjects, 28 years old | Randomized crossover study | 180 min (0, 10, 220, 30, 40, 60, 120, and 180 min) | A. Spaghetti and meat sauce plus lupin flour   Per kg body weight: 38.9 kJ, 0.3 g fat, 0.69 g protein, and 0.89 g carbohydrate   B. Spaghetti and meat sauce plus whey   Per kg body weight: 35.9 kJ, 0.21 g fat, 0.69 g protein, and 0.9 g carbohydrate   C. Spaghetti and meat sauce   Per kg body weight: 29.1 kJ, 0.21 g fat, 0.32 g protein, and 0.89 g carbohydrate  Standardized for carbohydrates per kg body weight | Glucose and insulin | AUC of glucose ↓* (compared with spaghetti and meat sauce) and no difference (compared with whey); AUC of insulin ↓ (compared with spaghetti and meat sauce) and ↓* (compared with whey) |

Supplementary table 1. Continued

| **Reference** | **Subjects** | **Study design** | **Postprandial period (time points)** | **Intervention** | **Relevant outcomes** | **Results intervention vs. control** |
| --- | --- | --- | --- | --- | --- | --- |
| Singh et al. (2024) ^37^ | 13 healthy subjects, 20 years old | Randomized crossover study | 120 min (0, 15, 30, 45, 60, 90, and 120 min) | A. Masala Vadai snack based on bengal gram (38 g) B. Medu Vadai snack based on black gram (26 g)   Snacks were standardized for 12.5 g available carbohydrates | Glucose | No significant differences between snacks (data for comparison with glucose solution not shown) |
| Thompson et al. (2012) ^34^ | 17 type 2 diabetic subjects, 58.6 years old | Randomized crossover study | 180 min (0, 30, 60, 90, 120, 150, and 180 min) | A. Pinto beans (177 g) and white rice   274 kcal, 0.4 g fat, 11.6 g protein, 59.7 g carbohydrate, and 10 g fiber   B. Black beans (115 g) and white rice   243 kcal, 0.8 g fat, 9.6 g protein, 55.5 g carbohydrate, and 5.8 g fiber   C. Kidney beans (139 g) and white rice   267 kcal, 0.4 g fat, 10.9 g protein, 58.7 g carbohydrate, and 9.1 g fiber   D. White rice   232 kcal, 0.5 g fat, 4.8 g protein, 49.5 g carbohydrate, and 0.7 g fiber  Standardized for available carbohydrates | Glucose | AUC of glucose ↓* (for pinto beans and black beans) and ↓ (for kidney beans) |

Supplementary table 1. Continued

| **Reference** | **Subjects** | **Study design** | **Postprandial period (time points)** | **Intervention** | **Relevant outcomes** | **Results intervention vs. control** |
| --- | --- | --- | --- | --- | --- | --- |
| Tsuchiya et al. (2022) ^44^ | 15 healthy subjects, 40.2 years old | Randomized crossover study | 180 min (0, 15, 30, 45, 60, 90, 120, and 180 min) | A. Yellow pea pasta (248 g)   1371 kJ, 1.9 g fat, 19.7 g protein, 50 g carbohydrate, and 14.7 g fiber   B. Wheat pasta   1637 kJ, 2 g fat, 12.9 g protein, 77 g carbohydrate, and 4.5 g fiber  Standardized for volume | Glucose, insulin, satiety, hunger, and fullness | AUCs of glucose and insulin ↓*; no significant difference in satiety, hunger, and fullness |
| Winham et al. (2017) ^52^ | 12 healthy women, 36 years old | Randomized crossover study | 120 min (0, 30, 60, 90, and 120 min) | A. Black beans (130 g) and white rice   263 kcal, 0.8 g fat, 11.2 g protein, 56.1 g carbohydrate, and 7.5 g fiber   B. Chickpeas (130 g) and white rice   258 kcal, 2.3 g fat, 9.2 g protein, 53.1 g carbohydrate, and 5.5 g fiber   C. White rice   232 kcal, 0.5 g fat, 4.8 g protein, 49.5 g carbohydrate, and 0.7 g fiber  Standardized for available carbohydrates (50 g) | Glucose and insulin | AUC of glucose ↓* (for chickpeas) and ↓ (for black beans); AUC of insulin ↑ (for chickpeas and black beans) |

Supplementary table 1. Continued

| **Reference** | **Subjects** | **Study design** | **Postprandial period (time points)** | **Intervention** | **Relevant outcomes** | **Results intervention vs. control** |
| --- | --- | --- | --- | --- | --- | --- |
| Winham et al. (2022) ^53^ | 18 healthy subjects, 23.5 years old | Randomized crossover study | 180 min (0, 30, 60, 90, 150, and 180 min) | A. Black bean pasta (knife mill; 239 g)   239 kcal, 6.5 g fat, 19.5 g protein, 72.1 g carbohydrate, and 22.1 g fiber   B. Whole black beans (197 g)   446 kcal, 5.4 g fat, 20.9 g protein, 78.2 g carbohydrate, and 28.2 g fiber   C. White bread   289 kcal, 4.3 g fat, 10.2 g protein, 52.6 g carbohydrate, and 2.6 g fiber  Standardized for available carbohydrates (50 g) | Glucose, insulin. appetite, satiation, and hunger | AUCs of glucose and insulin ↓* (for whole black beans) and ↓ (for black bean pasta); appetite and hunger ↓* and satiation ↑* (for black bean pasta and whole black beans) |

Supplementary table 1. Continued

| **Reference** | **Subjects** | **Study design** | **Postprandial period (time points)** | **Intervention** | **Relevant outcomes** | **Results intervention vs. control** |
| --- | --- | --- | --- | --- | --- | --- |
| Yoshimoto et al. (2020) ^43^ | 12 healthy subjects, 37.8 years old | Randomized crossover study | 120 min (0, 15, 30, 45, 60, 90, and 120 min) | A. Dehulled yellow pea pasta   1394 kJ, 2 g fat, 22 g protein, 50 g carbohydrate, and 12 g fiber   B. Unshelled yellow pea pasta   1425 kJ, 2 g fat, 21 g protein, 50 g carbohydrate, and 18 g fiber   C. White rice   917 kJ, 0 g fat, 3 g protein, 50 g carbohydrate, and 2 g fiber | Glucose and insulin | AUC of glucose ↓* (for dehulled and unshelled yellow pea pastas); AUC of insulin ↓* (for unshelled yellow pea pasta) and ↑ (for dehulled yellow pea pasta) |
| Zeinstra et al. (2019) ^63^ | 12 healthy subjects, 31.7 years old | Randomized crossover study | 180 min (15, 30, 45, 60, 75, 90, 120, 150, and 180 min) | A. Green peas (84 g) B. *Lemna minor* (duck wheat; 64 g) D. Standardized for protein (20 g) | Glucose, insulin, and amino acids | Amino acid concentration and insulin ↑* (for peas); glucose ↓* (for peas) |

Supplementary table 1. Continued

| **Reference** | **Subjects** | **Study design** | **Postprandial period (time points)** | **Intervention** | **Relevant outcomes** | **Results intervention vs. control** |
| --- | --- | --- | --- | --- | --- | --- |
| Zhu et al.  (2019) ^61^ | 10 healthy women, 20.7 years old | Randomized crossover study | 120 min (0, 20, 30, 45, 60, 90, and 120 min); 240 min for fullness | A. Adzuki beans (83 g) cooked for 40 or 70 min   277 kcal, 0.5 g fat, 18.4 g protein, 50 g carbohydrate, and 6.4 g fiber   B. White rice   222 kcal, 0.5 g fat, 4.5 g protein, 50 g carbohydrate, and 0.7 g fiber | Glucose and fullness | AUC of glucose ↓*; fullness ↑ |
| Zurbau et al. (2019) ^62^ | 17 healthy subjects, 26.7 years old | Randomized crossover study | 240 min (AIX: 0, 60, 120, 180, and 240 min; glucose: 15, 30, 45, 60, 90, 120, 180, and 240 min) | A. Chickpeas (113 g)   462 kcal, 14 g fat, 23 g protein, 50 g carbohydrate, and 20 g fiber   B. Large potato meal   424 kcal, 15 g fat, 22 g protein, 50 g carbohydrate, and 6 g fiber   C. Small potato meal   418 kcal, 26 g fat, 29 g protein, 17 g carbohydrate, and 2 g fiber   D. Pasta meal   462 kcal, 2 g fat, 17 g protein, 94 g carbohydrate, and 5 g fiber | Glucose and AIX | AUC of glucose ↓* (compared with large potato and pasta meals) and ↓ (compared with small potato meal); AIX at 4 h ↓* |

AIX, augmentation index; CCK, cholecystokinin; GIP, gastric inhibitory peptide; GLP-1, glucagon-like peptide 1; HOMA, Homeostasis Model Assessment Index; ORAC, oxygen radical absorbance capacity; PYY, peptide YY; sICAM1, soluble intercellular adhesion molecule-1; sVCAM1, soluble vascular cell adhesion molecule-1; TAG, triglyceride; TC, total cholesterol. * significant effects.
